# Addition of Aliskiren to Angiotensin Receptor Blocker Improves Ambulatory Blood Pressure Profile and Cardiorenal Function Better than Addition of Benazepril in Chronic Kidney Disease

**DOI:** 10.3390/ijms140815361

**Published:** 2013-07-24

**Authors:** Masato Ohsawa, Kouichi Tamura, Tomohiko Kanaoka, Hiromichi Wakui, Akinobu Maeda, Toru Dejima, Kengo Azushima, Kazushi Uneda, Ryu Kobayashi, Yuko Tsurumi-Ikeya, Yoshiyuki Toya, Tetsuya Fujikawa, Satoshi Umemura

**Affiliations:** Department of Medical Science and Cardiorenal Medicine, Yokohama City University Graduate School of Medicine, Yokohama 236-0004, Japan; E-Mails: t106008a@yokohama-cu.ac.jp (M.O.); to_kana8@yokohama-cu.ac.jp (T.K.); hiro1234@yokohama-cu.ac.jp (H.W.); maeda_chigasaki@yahoo.co.jp (A.M.); torudejima@yahoo.co.jp (T.D.); t116002f@yokohama-cu.ac.jp (K.A.); k_uneda@yokohama-cu.ac.jp (K.U.); t136034d@yokohama-cu.ac.jp (R.K.); ZVE04545@nifty.ne.jp (Y.T.-I.); ystoya@yokohama-cu.ac.jp (Y.T.); tftf@yokohama-cu.ac.jp (T.F.); umemuras@med.yokohama-cu.ac.jp (S.U.)

**Keywords:** albuminuria, ambulatory blood pressure, direct renin inhibitor, left ventricular hypertrophy, hypertension (kidney)

## Abstract

An altered ambulatory blood pressure (BP) and heart rate (HR) profile is related to chronic kidney disease (CKD) and cardiorenal syndrome. In this study, we examined the effects of aliskiren, when added to angiotensin II type 1 receptor blockers, on ambulatory BP and cardiorenal function in CKD. Thirty-six hypertensive CKD patients were randomly assigned to the aliskiren add-on group (*n =* 18) or the benazepril add-on group (*n =* 18). Ambulatory BP and cardiorenal function parameters were measured at baseline and 24 weeks after treatment. Compared with the benazepril group, nighttime systolic BP variability in the aliskiren group was lower after treatment. Albuminuria was decreased in the aliskiren group, but not in the benazepril group. In addition, left ventricular mass index (LVMI) was significantly lower in the aliskiren group than in the benazepril group after treatment. In the aliskiren group, multivariate linear regression analysis showed an association between changes in albuminuria and changes in nighttime systolic BP. Furthermore, there were associations between changes in LVMI and changes in daytime HR variability, as well as between changes in LVMI and changes in plasma aldosterone concentration. These results suggest that aliskiren add-on therapy may be beneficial for suppression of renal deterioration and pathological cardiac remodeling through an improvement that is effected in ambulatory BP and HR profiles.

## 1. Introduction

Hypertensive patients with chronic kidney disease (CKD) and diabetes are increasing in number, and cardiovascular complications are the most common cause of death in these hypertensive patients. Thus, it would be a considerable advance to elucidate the mechanisms involved in the renal deterioration and cardiovascular events associated with hypertension complicated by CKD and diabetes and to identify therapeutic approaches that inhibit these cardiorenal consequences. Recent evidence has indicated that ambulatory, as well as clinical blood pressure (BP) is important for a proper estimation of BP control. In particular, ambulatory BP monitoring has allowed an accurate diagnosis of hypertension [1,2] and determination of the BP and heart rate (HR) circadian rhythms under different pathophysiological conditions, including hypertension and CKD, and it may afford a more accurate prognosis than clinical BP measurement [3–5]. The circadian BP pattern in hypertensive patients with CKD has been found to exhibit a blunted nocturnal decrease in BP, which is associated with autonomic neuropathy and nephropathy [6,7]. The loss of nocturnal BP dipping has been considered to be a risk factor for the progression of nephropathy and to be of prognostic value with respect to target organ damage and cardiovascular morbidity in CKD patients [5,8–10].

Activation of the renin-angiotensin system (RAS) has been demonstrated to be involved in both the pathogenesis of CKD and its cardiovascular complications, and blockade of the RAS by angiotensin-converting enzyme inhibitors (ACEI) and angiotensin II (Ang II) type 1 receptor (AT1R)-specific blockers (ARB) has been shown to exert various protective effects on CKD progression and cardiovascular complications, at least partially, through a reduction in urinary protein/albumin excretion [11–14]. Both ACEI and ARB function downstream of the rate-limiting step in the RAS cascade, which involves the renin-catalyzed conversion of angiotensinogen to angiotensin I (Ang I). This, in turn, leads to the promotion of renin release and to an increase in plasma renin activity (PRA) via the intrarenal short feedback loop, due to the lack of the AT1R-mediated suppression of renin production in the juxtaglomerular cells of the kidney. Renin inhibition is a means of achieving RAS blockade. Aliskiren is a direct renin inhibitor that acts at the rate-limiting step of the RAS cascade, inhibiting the formation of Ang I from angiotensinogen. Therefore, unlike either ACEI or ARB, aliskiren does not induce a compensatory increase in PRA, but rather, reduces it [15,16].

Although in the “Aliskiren Trial in Type 2 Diabetes Using Cardiorenal Endpoints” (ALTITUDE) trial [17], which examined the additional effect of aliskiren to therapy with ACEI or ARB in patients with type 2 diabetes, who were at high risk for these complications, aliskiren did not show a benefit for renal and cardiovascular outcomes, with an increase in adverse effects; the effectiveness and safety of aliskiren add-on therapy for ambulatory BP and HR profiles in hypertensive patients with CKD is still unclear. Since aliskiren is reported to exert a long-lasting BP lowering effect in hypertension [18,19], we hypothesized that aliskiren exerts a beneficial influence on the ambulatory BP profile with concomitant cardiorenal protective effects, such as improvements in albuminuria and cardiac hypertrophy, in CKD. Therefore, in this study, we compared the therapeutic effect of aliskiren add-on therapy with those of ACEI (benazepril) add-on therapy in terms of the ambulatory BP and cardiorenal function in hypertensive patients with CKD.

## 2. Results

### 2.1. Patient Characteristics

Thirty-seven hypertensive patients with CKD were enrolled from November, 2009, to July, 2011. At the inclusion visit, one of them did not fulfill the selection criteria. The causes of CKD were hypertensive nephrosclerosis (*n =* 16), diabetic nephropathy (*n =* 13) and chronic glomerulonephritis (*n =* 7). Before participation in the study, written informed consent was obtained. The patients entered the run-in period and were randomized by a sealed envelope method to the aliskiren add-on group (*n =* 18) or the benazepril add-on group (*n =* 18). Table 1 shows the demographic and baseline characteristics of the participants. Any additional other treatments in both groups are shown in Table 2. During treatment, one patient from the aliskiren group (discontinuation, *n =* 1) and five patients from the benazepril group (adverse reaction, *n =* 4; consent withdrawal, *n =* 1) were lost to follow-up.

The aliskiren add-on therapy was well-tolerated in all of the patients, without any significant adverse events, and the average aliskiren dose was 176.5 ± 14.3 mg daily after a period of 24 weeks of treatment. On the other hand, four patients of the benazepril add-on group discontinued benazepril therapy, due to adverse events (cough, *n =* 3; hypotension, *n =* 1), and the average benazepril dose was 7.3 ± 0.7 mg daily after a period of 24 weeks of treatment. There were two patients of the aliskiren add-on group and one patient of the benazepril add-on group who had previously received ACE inhibitor and which had been converted to ARB for reasons unrelated to this study in these patients. The wash-out period had been for 69 weeks in one patient of the aliskiren add-on group and for 78 weeks in another patient. In one patient of the benazepril add-on group, the wash-out period had been for 13 weeks.

### 2.2. Effects of Aliskiren or Benazepril Add-On Therapy on the Clinic BP and Ambulatory BP Profiles

Since the results of this study showed that the aliskiren add-on therapy was better-tolerated than the benazepril add-on therapy, we analyzed the delta values (absolute values after the 24 weeks of the study period minus those at baseline), in addition to absolute values after the 24 weeks of the study period, to strictly compare the effects of anti-hypertensive treatment between the two groups.

Changes in clinic BP are shown in Table 3. Both the aliskiren and benazepril groups achieved the BP goal (BP < 130/80 mmHg), with no significant differences between groups (aliskiren *vs.* benazepril; systolic BP, −9.8 ± 1.8 *vs.* −13.1 ± 2.0, *p* = 0.226; diastolic BP, −6.9 ± 1.5 *vs.* −6.6 ± 1.5, *p* = 0.904). Systolic and diastolic BP did not differ between the two groups at any time point during the treatment.

Table 4 shows the 24 h, daytime and nighttime ambulatory BP and HR values, including their variability at baseline and after 24 weeks of treatment with aliskiren or benazepril. Ambulatory systolic/diastolic BP were comparably lowered in the aliskiren and benazepril groups after the 24-week treatment period (Table 4). The nighttime systolic short-term BP variability in the aliskiren group was significantly lower after 24 weeks of treatment compared with that in the benazepril group, although the absolute values of nighttime systolic short-term BP variability were increased in both groups (Table 4). Ambulatory HR and its variability were similar in the two groups after therapy.

### 2.3. Effects of Aliskiren or Benazepril Add-On Therapy on Markers of Renal Function, Cardiac Function and Oxidative Stress

At baseline, the eGFR and UACR did not differ significantly between the aliskiren and benazepril groups, and the eGFR after 24 weeks of treatment was comparable in the two groups (Table 5). On the other hand, the UACR after treatment was significantly decreased in the aliskiren group, but not in the benazepril group (Table 5). Pentosidine, which is a maker of oxidative stress, was similar in the aliskiren and benazepril groups (Table 5). In the echocardiographic findings, LVMI was significantly lower in the aliskiren group compared with the benazepril group after treatment (Table 6).

### 2.4. Univariate and Multivariate Linear Regression Analyses for Determination of Factors Contributing to Regression of Albuminuria and Amelioration of Cardiac Hypertrophy

To identify the factors affecting the beneficial changes in UACR and LVMI by aliskiren add-on therapy, we performed univariate and multivariate linear regression analyses in the aliskiren group. On univariate analysis, the changes in the UACR were positively correlated with the changes in the nighttime systolic BP (β = 0.642, *p* = 0.007) in the aliskiren add-on group. The changes in LVMI were negatively correlated with the changes in the daytime HR variability (β = −0.517, *p* = 0.049). Multivariate linear regression analyses in the aliskiren add-on group also indicated a significant association between changes in the UACR and the changes in the nighttime systolic BP (β = 0.612, *p* = 0.013) (Table 7). Furthermore, there was a significant association between the changes in the LVMI and changes in the daytime HR variability (β = −0.526, *p* = 0.029). The changes in the LVMI were also significantly associated with the changes in the plasma aldosterone concentration (β = 0.494, *p* = 0.038).

## 3. Discussion

The results of the present study showed that add-on treatment with either aliskiren or benazepril significantly decreased clinic BP in hypertensive CKD patients who had been already treated with ARB. However, the aliskiren add-on therapy decreased the residual albuminuria better than the benazepril add-on therapy, with concomitantly lower values for the nighttime short-term BP variability and LVMI after the treatment period. These data suggest the benefit of aliskiren therapy for hypertensive CKD patients results from an inhibition of renal injury and pathological cardiac remodeling, at least in part through an improvement in the short-term BP variability.

In the present study, the coefficients of variation (CV) in the BP and HR measured every 30 min was used as an index of short-term BP and HR variability to assess the circadian BP patterns and sympathovagal balance in hypertensive CKD patients, respectively. The increase in short-term BP variability and the decrease in HR variability may be partly explained by a disordered baroreflex function associated with an increased stiffness of the large elastic arteries and cardiac autonomic dysfunction. Previous studies have reported that short-term BP and HR variability on ambulatory BP monitoring is associated with cardiovascular disease in both hypertension and diabetes [3,20–22]. The results of our previous cross-sectional study also showed that short-term BP variability was significantly increased in CKD patients with coronary artery disease and, in particular, that an increase in nighttime short-term BP variability was strongly associated with the degree of left ventricular (LV) hypertrophy and the prevalence of coronary artery disease in CKD patients [23,24].

There is a well-established role for RAS in promoting hypertension- and CKD-related organ damage. Reducing proteinuria/albuminuria is critically important for the regression of CKD [25], and the evidence indicates that ARB and ACEI mainly reduce cardiovascular and renal risks by reducing proteinuria/albuminuria [11,14]. In our previous studies, the ARB-mediated preferential decrease in the nighttime BP level was accompanied by a reduction in albuminuria in CKD patients, without any long-term decrease in eGFR [7,26]. The results of the ACCOMPLISH study indicated that not only a reduction of albuminuria, but also a long-term preservation of eGFR is important for the suppression of CKD progression and cardiovascular complications [27].

With respect to the beneficial effects of aliskiren in CKD patients, aliskiren in addition to losartan treatment reportedly exerted a significant inhibitory effect on albuminuria, which was independent of its BP lowering effect in the “Aliskiren in the Evaluation of Proteinuria in Diabetes” (AVOID) study [28], and previous studies had already shown that aliskiren is effective and well-tolerated in Japanese CKD patients with hypertension [29,30]. Although there was a decreased nighttime systolic BP in the aliskiren add-on group compared with the benazepril add-on therapy, the changes in nighttime systolic BP between the aliskiren add-on group and the benazepril add-on group did not reach statistical significance with this number of patients. On the other hand, the results of the present study revealed that albuminuria were decreased in the aliskiren group with a concomitant association with nighttime systolic BP, but not in the benazepril group, thereby supporting the notion that the nighttime BP level might be one of the determinants of renal injury along with albuminuria. In a mouse model, the combination of a half dose of aliskiren and valsartan greatly reduced albuminuria, cardiac hypertrophy and tissue inflammation with attenuation of oxidative stress compared with monotherapy with a full dose of either drug, and those greater effects were independent of BP decrease [31]. Therefore, it would be possible that the parameters not examined in this study are associated with the reduction in UACR.

Furthermore, multivariate linear regression analysis showed a significant correlation between changes in LVMI and changes in HR variability in the aliskiren add-on group. Utilization of the ambulatory BP monitoring device, TM-2425, enabled us to assess HR variability during 24 h, daytime and nighttime periods. HR variability is mainly regulated by the cardiac sympathovagal balance, and it is affected by the relative sympathetic predominance that occurs via cardiac autonomic dysfunction. A lower HR variability has been associated with adverse cardiovascular outcomes in settings, such as post-myocardial infarction, coronary artery disease, congestive heart failure, diabetes and end-stage renal disease [22,32,33]. A recent study also showed that a lower HR variability occurs commonly in advanced stage CKD patients, due to cardiac autonomic neuropathy, and this is associated with increased cardiovascular complications and mortality in CKD patients, thereby suggesting an important role for HR variability in both the progression of CKD and the development of cardiorenal syndrome [34].

Furthermore, sympathetic predominance in CKD patients is reported to contribute to the development of cardiac hypertrophy despite antihypertensive treatment [35]. Since aliskiren was shown to reduce sympathetic nerve activity with BP lowering in CKD patients in a previous study [36], the results of the present study appear to indicate that aliskiren-mediated inhibition of sympathetic nerve activity, as revealed by the increase in HR variability in the aliskiren group, may be involved in the suppression of cardiac hypertrophy. Finally, multivariate linear regression analysis also showed a significant correlation between changes in LVMI and changes in the plasma aldosterone concentration. The results of the “Aliskiren in Left Ventricular Hypertrophy” (ALLAY) study showed that the aliskiren-mediated suppression of aldosterone is involved in the regression of cardiac hypertrophy in hypertension [37], which would be consistent with the results of the present study.

There were several limitations to this study. Firstly, our sample size was relatively small to determine significance. In addition, although albuminuria was measured once on each occasion in this study, UACR is reported to be highly variable. Furthermore, the 24-week intervention period is relatively short, and it may be argued that the reduction in albuminuria and the regression of LVH during the short period may not improve long-term renal and cardiovascular outcomes. Although the addition of aliskiren to the ARB resulted in an additional reduction in proteinuria in CKD patients in the present study and those with diabetic nephropathy in the AVOID study [28], an increase in adverse events, such as hypotension and hyperkalemia, and no apparent benefits among patients randomized to aliskiren in the ALTITUDE trial, prompted early study termination [17]. In addition, the combination therapy with telmisartan and ramipril reduced UACR to a greater extent than ramipril monotherapy, but it worsened the decline in eGFR with an increased risk of hypotension, hyperkalemia and acute renal impairment, especially in the subgroup with no history of hypertension, in *post hoc* analysis of ONTARGET [38]. Therefore, these dual RAS blockades with the combination of the highest dose of RAS inhibitors in high-risk patients without hypertension might increase hypotension and not show beneficial effects in these studies.

On the other hand, aliskiren has been reported to exert longer-lasting and more potent renal vasodilation than what is achieved with ARB and ACEI [39]. Furthermore, the results of an animal study showed that aliskiren can reduce the renal expression of the (pro)renin receptor to efficiently block the prorenin-induced activation of renal RAS, which would be a different additional beneficial effect from that of ARB and ACEI [40]. Therefore, the long-term organ protective potential of aliskiren and its putative superiority over existing therapies remains to be elucidated. Further studies are also necessary to compare the beneficial effects of aliskiren-based therapy on target organ function with those of ACEI- or ARB-based therapy in hypertension and CKD. Our results suggest that aliskiren add-on therapy may exert its cardiorenal protective effects, at least partly, through the improvement in ambulatory BP and HR profiles and the suppression of aldosterone in hypertensive CKD patients.

## 4. Experimental Section

### 4.1. Study Design

This was a randomized, open-label and parallel-group study (UMIN Clinical Trials Registry: UMIN000002546) and was conducted at the outpatient clinic of the Department of Internal Medicine, Yokohama City University Hospital (Yokohama, Japan). The study consisted of a 2-week run-in period and a 24-week active treatment period. This study was approved by the Ethics Committees of Yokohama City University Hospital, and written informed consent was obtained from every participant in accordance with the Declaration of Helsinki.

The primary outcomes were the comparison of the changes in UACR and LVMI from baseline to after 24 weeks of treatment between the addition of aliskiren and benazepril. Secondary outcomes were the comparison of the changes in ambulatory BP and HR profiles, oxidative stress and RAS components between the addition of aliskiren and benazepril.

### 4.2. Study Participants

CKD patients were eligible for the study if they were ≥20 years (or older), with mild-to-moderate hypertension (clinic systolic BP ≥ 130 mmHg and/or diastolic BP ≥ 80 mmHg) and albuminuria stages A2 or A3 (urinary albumin excretion rate, UACR ≥ 30 mg/g-creatinine), despite the preceding antihypertensive therapy with ARB at the standard dose for a period of more than 4 weeks. CKD was diagnosed by the presence for more than 3 months of albuminuria (urinary albumin excretion rate, UACR ≥ 30 mg/g-creatinine), proteinuria (urinary protein excretion rate, UPCR ≥ 150 mg/g-creatinine) or eGFR < 60 mL/min/1.73 m^2^). We calculated the eGFR using a revised equation for the Japanese population: eGFR (mL/min/1.73 m^2^) = 194 × serum creatinine^−1.094^ × Age^−0.287^ × 0.739 (if female) [41]. The exclusion criteria included severe hypertension (clinic systolic BP ≥ 180 mmHg and/or diastolic BP ≥ 110 mmHg), patients who were on dialysis or taking immunosuppressants, women who were nursing or pregnant, clinically significant heart disease, stroke, renal artery stenosis, hepatic dysfunction and known hypersensitivity to any ingredient in the study medications.

### 4.3. Study Treatment

After the run-in period, along with the continuation of any standard dose ARB, eligible patients were randomized either to the aliskiren or the benazepril add-on group. Patients in the aliskiren add-on group were initially given 150 mg of aliskiren once daily in the morning, with the dose of aliskiren titrated up to 300 mg daily, as needed, during the 24-week active treatment period. Patients in the benazepril add-on group were initially given 2.5 mg of benazepril once daily in the morning, with the dose of benazepril titrated up to 10 mg daily as needed during the 24-week active treatment period. In both groups, the antihypertensive therapy was aimed at achieving the BP goal (BP < 130/80 mmHg). The dose of ARBs and anti-hyperglycemic drugs before the start of the trial were not changed during the treatment period.

### 4.4. Clinical BP and Ambulatory BP Monitoring

The clinical BP was measured at trough (24 ± 2 h post-dose) using a calibrated standard mercury sphygmomanometer and the recommended cuff sizes in a sitting position [42]. Two measurements were taken at 1- to 2-min intervals, and their average was used to calculate the clinic BP.

The ambulatory BP and HR were monitored every 30 min with a fully automated device (TM-2425, A&D, Tokyo, Japan), essentially as described previously [23,24,26,43–45]. The ambulatory BP and HR monitoring was repeated in patients who had >20% values missing, >30% error rate for the total readings or values missing for more than two consecutive hours. The following readings were omitted because of technical artifacts: systolic BP > 250 mmHg or <70 mmHg, diastolic BP > 130 mmHg or <30 mmHg, pulse pressure > 160 mmHg or <20 mmHg, systolic differences > 60 mmHg or diastolic differences > 30 mmHg compared with the immediately preceding or subsequent values. The patients were instructed to fill out a diary to record the time of sleeping, rising and other daytime activities. Therefore, the terms “daytime” and “nighttime” in the present study reflect the average period during which the subjects were awake/upright and asleep/supine, respectively.

Short-term BP variability, which is composed of coefficients of variation (CV) of the BP values obtained from ambulatory BP monitoring, is defined as the within-subject SD of all of the systolic and diastolic readings at 30-min intervals divided by the mean BP during the course of the measurement periods. HR variability, which is composed of the CV of HR values, is defined as the within-subject SD of all of the HR values at 30-min intervals divided by the mean HR [23,24,26,43–45].

### 4.5. Laboratory Measurements

Venous blood and urine samples were drawn and collected in the morning after an overnight fast. Blood sampling for the parameters of the RAS components was performed after the patients had spent 30 min at quiet rest in a recumbent position. In plasma, the PRA, active renin concentration (ARC) and aldosterone concentration were measured by radioimmunoassay. Plasma pentosidine was determined using an ELISA kit (SRL, Tokyo, Japan). Other parameters were measured by routine methods in the Department of Clinical Chemistry, Yokohama City University School Hospital.

### 4.6. Echocardiography

Echocardiographic studies were performed by trained echocardiographers blinded to the treatment assignment, and parameters of left ventricular (LV) function and hypertrophy, including the LV mass index (LVMI), were measured and calculated, as described previously [43].

### 4.7. Statistical Analysis

Data were expressed as the mean ± SE or as a percentage. To examine the effects of anti-hypertensive treatment, the delta values (absolute values after the 24 weeks of the study period minus those at baseline), in addition to absolute values after the 24 weeks of the study period, were used for analysis. Significant differences between the groups were assessed by unpaired *t*-test or nonparametric analysis using the Wilcoxon U test for variables that were not normally distributed. Differences between the groups for categorical variables were analyzed using the Chi-square test. A repeated-measures ANOVA model utilized clinic BP and HR obtained during the 24 weeks of treatment. Univariate and multivariate linear regression analyses were performed to identify the factors affecting the changes in UACR and LVMI. The independent variables entered into the multivariate models were those that were significantly associated in univariate analyses or were significantly different between the groups after the treatment. The *post hoc* estimated statistical power was >95% to detect differences in changes of UACR and LVMI between the aliskiren add-on group and the benazepril add-on group. SPSS18.0 statistical software was used for statistical analysis. A value of *p* < 0.05 was considered significant.

## 5. Conclusions

In summary, the results of the present study suggested that aliskiren add-on therapy is beneficial for the suppression of renal deterioration and pathological cardiac remodeling though the improvement in ambulatory BP and HR profiles and the suppression of aldosterone in hypertensive CKD patients with albuminuria.

## Figures and Tables

**Table 1:** Demographic characteristics of the study groups at baseline.

	Benazepril (*n =* 18)	Aliskiren (*n =* 18)	*p*-value
Age (years)	67.1 ± 2.7	65.2 ± 3.3	0.647
Gender (female/male)	5/13	6/12	0.717
Body mass Index (kg/m^2^)	24.5 ± 1.0	25.6 ± 1.2	0.591
Waist size (cm)	89.0 ± 2.4	92.8 ± 2.5	0.457
Current smoking (n (%))	4(22)	4(22)	0.655
Diabetes mellitus (n (%))	9(50)	11(61)	0.502
Dyslipidemia (n (%))	14(78)	14(78)	0.655
Cerebrovascular disease (n (%))	2(11)	1(6)	0.500
Ischemic heart disease (n (%))	1(6)	1(6)	0.757
Cause of CKD (n (%))			0.500
Diabetic nephropathy	6(33)	7(39)	
Chronic glomerulonephritis	4(22)	3(17)	
Nephrosclerosis	8(44)	8(44)	
Systolic blood pressure (mmHg)	140 ± 2	140 ± 3	0.918
Diastolic blood pressure (mmHg)	81 ± 2	81 ± 2	0.985
Heart rate (beats/min)	71 ± 2	70 ± 2	0.637
HbA_1C_ (%)	6.4 ± 0.2	6.7 ± 0.3	0.267
LDL cholesterol (mg/dL)	98 ± 7	111 ± 6	0.110
HDL cholesterol (mg/dL)	56 ± 4	60 ± 4	0.311
Triglyceride (mg/dL)	131 ± 24	151 ± 19	0.174
Serum creatinine (mg/dL)	1.4 ± 0.2	1.5 ± 0.2	0.797
Estimated GFR (mL/min per 1.73 m^2^)	45.7 ± 4.7	46.3 ± 5.6	0.864
GFR stages (n (%))			0.340
G1	1(6)	2(11)	
G2	2(11)	3(17)	
G3a	3(17)	2(11)	
G3b	8(44)	6(33)	
G4	3(17)	4(22)	
G5	1(6)	1(6)	
Urinary albumin/creatinine ratio (mg/g Cr)	1015 ± 347	1885 ± 647	0.192
Albuminuria stages (n (%))			0.480
A1	0(0)	0(0)	
A2	7(39)	5(28)	
A3	11(61)	13(72)	
Serum potassium (mEq/L)	4.2 ± 0.1	4.2 ± 0.1	0.493

Values are means ± SE or number (percentage). CKD, chronic kidney disease; LDL, low-density lipoprotein; HDL, high-density lipoprotein; GFR, glomerular filtration rate.

**Table 2:** Medication in the study groups at baseline.

	Benazepril (*n =* 18)	Aliskiren (*n =* 18)	*p*-value
Antihypertensive agents (*n* (%))			
Angiotensin II receptor blockers	18(100)	18(100)	–
Angiotensin-converting enzyme inhibitors	0(0)	0(0)	–
Calcium-channel blockers	12(67)	14(78)	0.457
Thiazide diuretics	6(33)	6(33)	1.000
Loop diuretics	2(11)	5(28)	0.201
β-blockers	5(28)	5(28)	1.000
α-blockers	2(11)	3(17)	0.500
Central sympatholytic agents	1(6)	1(6)	0.757
Glucose-lowering agents (n (%))			
Insulin and insulin analogues	4(22)	4(22)	0.655
Sulfonylureas	2(11)	4(22)	0.329
α-glucosidase inhibitors	2(11)	3(17)	0.500
Thiazolidinediones	1(6)	1(6)	0.757
Dipeptidyl peptidase IV inhibitors	1(6)	0(0)	0.500
Lipid-lowering agents (n (%))			
Statins	11(61)	11(61)	1.000
Fibrates	0(0)	1(6)	0.500
Antiplatelet agents (n (%))	3(17)	2(11)	0.500

Values are number (percentage).

**Table 3:** Clinical BP and HR profile before and after add-on anti-hypertensive treatment.

Clinical	Benazepril			Aliskiren		
	
	Baseline	24 weeks	Δ	Baseline	24 weeks	Δ
Systolic BP (mmHg)	142 ± 2	128 ± 3	−13 ± 2	139 ± 2	129 ± 2	−10 ± 2
Diastolic BP (mmHg)	82 ± 2	76 ± 1	−7 ± 1	81 ± 2	74 ± 2	−7 ± 2
HR (beat/min)	70 ± 3	71 ± 2	0 ± 2	70 ± 2	70 ± 2	0 ± 2

Values are means ± SE; BP, blood pressure; HR, heart rate.

**Table 4:** Ambulatory BP and HR profile before and after add-on anti-hypertensive treatment.

	Benazepril			Aliskiren		
		
	Baseline	24 weeks	Δ	Baseline	24 weeks	Δ
Daytime						
Systolic BP (mmHg)	144 ± 3	137 ± 6	−7 ± 4	143 ± 3	136 ± 4	−6 ± 4
Diastolic BP (mmHg)	84 ± 3	80 ± 3	−3 ± 3	80 ± 2	78 ± 2	−2 ± 2
HR (beat/min)	70 ± 3	73 ± 3	3 ± 2	71 ± 2	71 ± 2	0 ± 2
Systolic BP variability (%)	12.0 ± 0.8	14.0 ± 1.2	2.0 ± 1.1	13.1 ± 0.6	14.2 ± 0.8	1.1 ± 0.9
Diastolic BP variability (%)	12.5 ± 1.1	14.7 ± 1.4	2.2 ± 1.8	13.5 ± 0.6	14.9 ± 0.8	1.4 ± 0.9
HR variability (%)	15.6 ± 1.4	14.4 ± 1.1	−1.2 ± 1.9	17.9 ± 1.2	17.7 ± 1.5	−0.2 ± 1.3

Nighttime						
Systolic BP (mmHg)	127 ± 4	127 ± 5	0 ± 4	132 ± 5	125 ± 4	−7 ± 4
Diastolic BP (mmHg)	73 ± 3	73 ± 3	0 ± 2	74 ± 3	71 ± 3	−3 ± 2
HR (beat/min)	62 ± 2	65 ± 3	3 ± 2	62 ± 2	63 ± 2	1 ± 1
Systolic BP variability (%)	10.3 ± 0.8	12.8 ± 1.4	2.5 ± 1.1	9.2 ± 0.6	9.6 ± 0.7 *	0.5 ± 0.9
Diastolic BP variability (%)	11.4 ± 1.3	13.0 ± 1.4	1.6 ± 1.3	10.8 ± 0.7	11.3 ± 0.8	0.5 ± 0.9
HR variability (%)	7.8 ± 1.1	9.3 ± 1.4	1.4 ± 1.2	9.2 ± 0.7	8.7 ± 1.0	−0.5 ± 1.3

Values are means ± SE; BP, blood pressure; HR, heart rate;

* *p* < 0.05 *vs.* benazepril group.

**Table 5:** Comparison of the effects of add-on anti-hypertensive treatments on parameters of renal function, oxidative stress and RAS components.

	Benazepril			Aliskiren		
		
	Baseline	24 weeks	Δ	Baseline	24 weeks	Δ
Estimated GFR (mL/min/1.73 m^2^)	43.1 ± 4.3	40.1 ± 4.6	−2.9 ± 1.8	48.2 ± 5.6	46.1 ± 5.6	−2.1 ± 0.9
UACR (mg/g Cr)	1309 ± 456	1340 ± 445	32 ± 131	1824 ± 683	1433 ± 589	−391 ± 135 *
Pentosidine (nm/L)	40.7 ± 5.0	36.7 ± 5.7	−4.0 ± 6.9	32.4 ± 2.8	32.3 ± 3.9	−0.1 ± 3.0
PRA (ng/mL/h)	1.9 ± 0.5	3.2 ± 1.6	1.3 ± 1.7	2.0 ± 0.5	0.5 ± 0.2 *	−1.5 ± 0.5 *
ARC (pg/mL)	18.3 ± 4.4	46.8 ± 32.4	28.5 ± 33.2	19.6 ± 3.7	136.0 ± 32.8 *	116.4 ± 31.2 **
PAC (pg/mL)	55.9 ± 10.9	49.3 ± 9.7	−6.5 ± 8.3	81.3 ± 17.1	68.4 ± 11.7	−13.0 ± 6.7
Serum potassium (mEq/L)	4.2 ± 0.2	4.2 ± 0.2	0.0 ± 0.2	4.2 ± 0.1	4.3 ± 0.1	0.1 ± 0.1

Values are means ± SE. RAS, renin-angiotensin system; GFR, glomerular filtration rate; UACR, urinary albumin/creatinine ratio; PRA, plasma renin activity; ARC, active renin concentration; PAC, plasma aldosterone concentration.

* *p* < 0.05 *vs.* benazepril group;

** *p* < 0.01 *vs.* benazepril group.

**Table 6:** Cardiac function parameters before and after add-on anti-hypertensive treatment.

	**Benazepril**			**Aliskiren**		
		
	Baseline	24 weeks	Δ	Baseline	24 weeks	Δ
LVMI (g/m^2^)	156 ± 9	157 ± 11	2 ± 7	141 ± 12	131 ± 10 *	−10 ± 5
FS (%)	42.1 ± 2.2	42.9 ± 2.6	0.8 ± 1.3	40.9 ± 1.9	41.2 ± 1.6	0.4 ± 2.0
EF (%)	72.2 ± 2.6	73.0 ± 3.0	0.8 ± 1.5	71.0 ± 2.2	71.6 ± 1.9	0.6 ± 2.2

Values are means ± SE; LVMI, left ventricular mass index; FS, fractional shortening; EF, ejection fraction;

* *p* < 0.05 *vs.* benazepril group.

**Table 7:** Multivariate linear regression analyses of factors associated with changes in UACR and LVMI in the aliskiren add-on group.

Variable	β	*p*-value
Change in UACR (mg/g Cr)		
Change in nighttime systolic BP (mmHg) (Model *R*^2^ = 0.475)	0.612	0.013
Change in LVMI (g/m^2^)		
Change in daytime HR variability (%)	−0.526	0.029
Change in plasma aldosterone concentration (pg/mL) (Model *R*^2^ = 0.519)	0.494	0.038

*R*^2^ = coefficient of determination. UACR, urinary albumin/creatinine ratio; LVMI, left ventricular mass index; BP, blood pressure; HR, heart rate.
